# An uncommon malignant cutaneous squamomelanocytic tumor

**DOI:** 10.3892/etm.2013.882

**Published:** 2013-01-04

**Authors:** HONG-YAN WANG, XUE-BIN ZHANG, RU-JUAN SU, CHUN-BAO WANG, XI LIU

**Affiliations:** Department of Pathology, First Affiliated Hospital, Medical College of Xi’an Jiaotong University, Xi’an, Shaanxi 710061, P.R. China

**Keywords:** squamomelanocytic tumor, solar keratosis, solar lentigo, histogenesis

## Abstract

The intermingling of two malignant neoplasms within the same cutaneous tumor is rare. No consensus has been reached for a clear definition and categorization. In the current study, we describe a cutaneous neoplasm; a squamomelanocytic tumor (SMT) with histological features combining those of a squamous cell carcinoma (SCC) and a malignant melanoma (MM). This is the second report of an oculocutaneous SMT, and concerns a subject with a 20-year history of solar lentigo coexisting with solar keratosis in the primary lesion. This type of tumor is quite rare, with a distinct cytological architecture and immunohistochemical features, and the differential diagnosis of SMT may be considered. However, the histogenesis, biological behavior and malignant potential of SMTs remain a matter of speculation. With regard to the treatment, complete surgical resection and close follow-up are recommended.

## Introduction

Despite the common occurrence of cutaneous melanoma and squamous cell carcinoma (SCC), only a few cases of a solitary skin tumor with an admixture of the features of melanoma and SCC have been reported. This tumor was first described by Rosen *et al*(1) and named as a squamomelanocytic tumor (SMT) by Pool *et al*(2). Boyd and Rapini (3), who reviewed 69 cutaneous collision tumor epidemiological studies, identified no cases of SMT. There are only eighteen reported cases of SMT in the literature. The most common anatomical locations are on the face and neck. Our case, the second report of oculocutaneous SMT, is a 63-year-old female, with solar keratosis and solar lentigo in the periphery. This is the first case of such a specific growth pattern. In a number of cases, it has been reported that the histogenesis of the SMT may be associated with burn scars and solar damage. However, in reference to our case, we consider that a long-term history of solar keratosis is correlated with the SMT, which may be the last stage of the solar keratosis process. Other cutaneous neoplasms, including a mixture of malignant melanoma (MM) with keratoacanthoma, SCC, pigmented squamous cell carcinoma, pigmented keratinizing basal cell carcinoma and pseudoepitheliomatous, are further discussed. A review of the literature and a case analysis for the potential histogenesis of SMT and histological differential diagnoses are discussed. This study was conducted in accordance with the Declaration of Helsinki and with approval from the Ethics Committee of the First Affiliated Hospital, Medical College of Xi’an Jiaotong University. Written informed consent was obtained from the participant.

## Case report

### Clinical presentation

A previously healthy 63-year-old Chinese female presented with a brown-coloured spot located on the left lateral canthus. The spot had been 2 mm in diameter for 20 years; however, it progressively expanded over six months to a dimension of 12x10x5 mm. One month later, a clinical diagnosis of MM or a pigmented basal cell carcinoma was considered; therefore, the lesion was completely excised. Due to the unusual epithelioid features of the tumor, the case was referred to the Department of Clinical Pathology, First Affliated Hospital of Xi’an Jiaotong University for a secondary consultation.

### Pathological features

**Figure 1.**
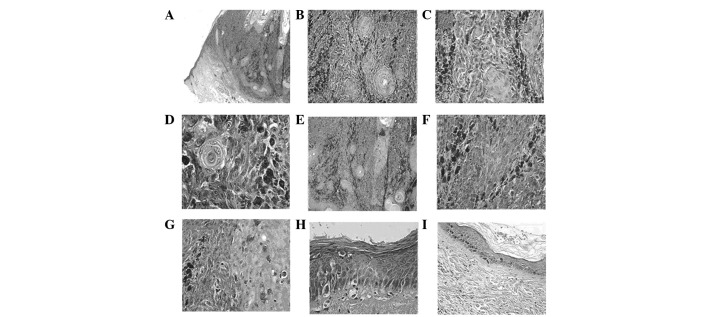
(A) A well-demarcated expansive nodule at the dermal-epidermal junction and within the dermis (original magnification, ×2). (B) Two different neoplastic cell proliferations were observed intermingling to exhibit a combination of nested growth patterns (original magnification, ×10). (C) The squamous component consisted of thin anastomosing epithelial cords and small whorled nests (original magnification, ×10). (D) Slight atypical squamous cells contained abundant eosinophilic cytoplasm and large, often hyperchromatic and vesicular nuclei (original magnification, ×40). (E) Epithelial proliferation formed a branch-like shape extending into the dermis and the basal layer was absent (original magnification, ×20). (F) Irregularly shaped nest of predominant atypical pigmented epithelioid cells (original magnification, ×20). (G) Single atypical melanocyte dispersed in the epidermis and mitotic figures were observed (original magnification, ×20). (H) Atypical epidermic basal cells lost polarity and presented larger, hyperchromatic and pleomorphic nuclei with mitosis (original magnification, ×40). (I) A lentigo was present with increased melanin in the basal layer (original magnification, ×10). Hematoxylin and eosin staining.

Low-power examination revealed a well-demarcated expansive nodule at the dermal-epidermal junction and within the dermis (Fig. 1A). High-power examination revealed that the tumor was composed of two cell types. The two neoplastic cell proliferations were discerned intermingling within the epidermis and dermis (Fig. 1B). The first cell type observed consisted of slight atypical squamous cells with abundant eosinophilic cytoplasm and large, often hyper-chromatic and vesicular nuclei. Squamous pearls, squamous cysts, dyskeratotic cells, apoptotic bodies and mitotic figures were also observed. The squamous cell component consisted of thin anastomosing epithelial cords and small whorled nests, diffusely and irregularly distributed through the entire tumor, extending to the deep reticular dermis. The formation of papilla by overlying squamous epithelial proliferation was accompanied by hyperkeratosis and dyskeratosis. The continuous sections presented an area in which the epithelial proliferation formed a branch-like shape extending into the dermis. The basal layer was absent (Fig. 1C–E). The second cell type observed was an irregularly shaped nest of predominant atypical pigmented epithelioid cells (melanocytes). The cells had marginal ambiguity with little eosinophilic cytoplasm, in addition to plump to elongated spindle nuclei, with one or two predominant nucleoli. The mitotic cell count was ∼9 mitoses per 10 high-power fields. A number of cells had fine granular, brown to gray cytoplasmic granules of melanin, primarily arranged in small to large nests at the dermal-epidermal junction and proliferating to the deep reticular dermis; however, they did not extend to the hair follicles or eccrine gland. Single cells invading the epidermis were identified. (Fig. 1F and G). A significant amount of inflammatory and lymphocyte infiltrate was identified in the stroma.

Chronic solar keratosis was observed near the tumor and disruption of collagenous fibers was observed under the dyskeratotic epidermis, consistent with the chronic solar damage. Atypical epidermic basal cells had lost polarity and presented larger, hyperchromatic and pleomorphic nuclei with mitosis (Fig. 1H).

A solar lentigo was present beside the area of solar keratosis. Increased melanin and melanocytes in the basal layer, as well as abnormal basal cells manifesting a benign process and lentigo maligna existed in the adjacent epidermis (Fig. 1I).

### Immunohistochemistry and specific staining

**Figure 2.**
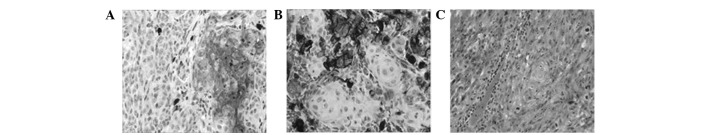
(A) Immunohistochemistry revealed diffuse cytoplasmic reactivity for pancytokeratin (original magnification, ×20). (B) Antibodies directed to the nucleus and cytoplasm showed reactivity for HMB-45 (original magnification, ×10). (C) Specific staining for melanin expression revealed that melanin had disappeared from the tumor cells (original magnification, ×20). HMB45, Melanoma Marker.

Immunohistochemistry revealed diffuse cytoplasmic reactivity for pancytokeratin and high molecular weight cytokeratin (HMWCK), and local cytomembrane reactivity for epithelial membrane antigen (EMA) and low molecular weight cytokeratin (LMWCK), in all areas revealing histopathological features of SCC (Fig. 2A). Antibodies directed to the nucleus and cytoplasm demonstrated reactivity for S-100 protein, and Melanoma Marker (HMB45) and Melan-A antibody reactivity demonstrated a strong positivity for the atypical melanocytic component (Fig. 2B). No subset of the tumor cells demonstrated combined staining for epithelial and melanocytic markers. The melanin staining identified that melanin had disappeared from the tumor cells, which excluded a differential diagnosis of other pigmentation diseases (Fig. 2C). Comparison of the mitotic activity in the two cellular elements revealed that ∼40% melanocytic cells and ∼5% squamous cells were positive for the proliferation marker, Ki-67.

Based on the histopathological findings and the immunohistochemical profile, the diagnosis of an SMT (12 mm in depth) was made.

## Discussion

SMTs are rare and they each have an identical growth pattern (1). The tumors characteristically contain an expansile, lobular, well-circumscribed nodule arising from the epidermis or from the deep reticular dermis. The Breslow depth range is 10–27 mm, with or without connection to the epidermis (1,2,4–11). The lesions may be infiltrative or have a tongue-like extension into the adjacent normal tissue, including vessels and hair follicles. The tumor demonstrates an intimate admixture of melanocytic and squamous cells. Immunohistochemistry indicates two clear populations: an atypical melanocytic component expressing S-100 and HMB-45 and atypical squamous cells expressing cytokeratin. High-index staining of cellular proliferation with Ki-67 ranged from 20 to 50%, for different components.

By reviewing the 18 previously documented cases (Table I), we identified that the patient age range was 32–94 years and only 6 cases occurred in individuals aged ≤55 years (2,7,8,12). No predominant gender predilection has been noted for SMT variants. Two cases of SMT occurred in patients with burn scars from their childhood, indicating that this stimulating condition increases the risk of developing malignancies. The most common anatomical locations are the head (9 cases, including forehead, eyebrow, canthus, temple, nose and ear), trunk (3 cases, including shoulder, scapula and back) and the extremities (6 cases, including thigh and leg). Our case concerned a 63-year-old female with a lesion in the face, combined with solar keratosis and solar lentigo with SMT. This is the first reported case with such a specific growth pattern.

The histological differential diagnoses of an SMT include: i) Collision tumors of MM with keratoacanthoma and SCC: the clear border and distinct growth pattern of epithelial neoplasms, including keratoacanthoma and SCC, even if they occasionally collide with MM, allow easy exclusion. The craterform appearance of keratoacanthoma presents the benign differentiation of cells without malignant features. However, SMTs present an intimate admixture of melanocytic and squamous cells, in contrast to collision tumors in which there are distinctly separate components of melanocytic and epithelial cells. ii) Pigmented SCC: a rare variant of SCC that consists of distinct malignant epithelial components admixed with benign dendritic melanocytes. SMTs present the coexistence of two malignant tumors intermingling in the same histological specimen. The characteristic immunohistochemical features of SMT are the expression of S-100 protein and positivity to HMB-45 and Melan-A antibodies for the atypical melanocytic components and cytokeratin positivity for the atypical squamous cells, while epithelial cells in pigmented SCC are negative for HMB-45 and S-100 protein (13). iii) Pigmented keratinizing basal cell carcinoma: this tumor comprises basal cell carcinoma and benign dendritic melanocytes. The basal cells are arranged in cords and nodules containing peripherally palisaded cells and have focal areas of squamous differentiation. Immunohistochemistry reveals epithelial components negative for HMB-45 and S-100. iv) MM with pseudoepitheliomatous hyperplasia: reactive epithelial pseudoepitheliomatous hyperplasia has been described as a reactive phenomenon in a series of MMs (14). Apart from abnormal serrated architecture and cytological features characteristic of MM, the hyperplastic epithelium lacks nuclear atypia, mitoses and prominent dyskeratosis. Furthermore, the melanocytic and the squamous components have distinctive cytological atypia and Ki-67 proliferation marker expression, consistent with malignancy.

The exact histogenesis of SMT has yet to be elucidated. Although a number of hypotheses have been proposed, no conclusive explanation been confirmed.

Rongioletti *et al* favored the hypothesis that a tumor in one cell type is colonized by a population of a second cell type (11). In the case reported by the authors, the pathological features suggest that the epithelial tumor represents an atypical solid-cystic hidradenoma colonized by the S-100/HMB-45 melanocytic malignant component. Another hypothesis is that these tumors represent biphenotypic malignant populations from a common precursor. Rosen *et al*(1) reported a case showing staining for S-100 protein and keratin in the same tumor cells. We did not identify biphenotype (combined S-100/cytokeratin positivity in the same cells) in our case.

Pool *et al*(2) interpreted that true malignant proliferation of two distinct phenotypes occurs as a result of close paracrine interactions. The authors considered that SMT is an unusual melanoma variant, demonstrating divergent epithelial differentiation.

Four categories of tumor histogenesis are considered: i) collision tumor, ii) colonization tumor, iii) combined tumor and iv) biphenotypic tumor.

We favor the hypothesis that SMT is associated with solar keratosis, and occurs as the final stage in this process. Epidemiological, clinicalpathological and molecular evidence indicates that solar keratosis represents an early stage in a biological continuum that ranges from carcinoma *in situ* to invasive SCC and primarily develops on sun-exposed skin. In a previous study, the prevalence of solar keratosis in the face and forehead was reported to be 32% in females and 13% in males (15). This indicates that solar keratosis may vary according to gender. Our study presents an elderly female with an oculocutaneous lesion. The continuous sections revealed an area of epithelial proliferation into the dermis. This lesion may initiate SCC origin from the epidermis, which may be a stage in the process of malignant solar keratosis.

Ultraviolet radiation stimulation of the skin and surgery in nevus-prone individuals are two causes of melanoma development. However, the presence of solar keratosis has been reported as a risk factor for melanoma, particularly for primary sun-exposed sites (15). An epidemiological study of 159 individuals, each with at least one area of solar keratosis, revealed that 39% of cases with melanoma were in older patients. Niamh *et al*(16) reported a case with a 2-year history of solar keratosis in the primary lesion. Two cases demonstrating that long-term sun exposure causes SMT have been reported (4,5). A case arising from lentigo maligna was reported by Pool *et al*(2). In our case, following a 20-year history of colored spots considered to be solar lentigines, SMT was diagnosed. Solar keratosis was also observed in the periphery of the focus and lentigo maligna was in existence in the adjacent epidermis. This indicates that SCC and MM are closely related to solar keratosis. In our case, we confirmed that solar keratosis induced SCC. Although the process of MM is not clear, we identified the histogenesis of SMT to be the final stage of solar keratosis.

The biological behavior of this unique combined tumor remains obscure. SCC and MM are capable of metastasis and mortality. MM are responsible for ∼7,300 mortalities each year, while between 1,300 and 2,300 individuals succumb each year as a result of nonmelanoma skin cancer, primarily metastatic SCC (17). Of the surviving cases in the present study, all have undergone clinical treatment and complete excision of the tumors with adequate margins, with a mean follow-up time of 31 months. Two studies have reported regional metastasis and there have been two reports of mortality as a result of metastatic melanoma (4,5). A longer follow-up and additional studies are required to validate the findings presented here and to elucidate the prognosis and most suitable therapeutic approach for combined cutaneous tumors.

## Figures and Tables

**Table I.** Squamomelanocytic tumors reported in the literature.

Author	Age (years)	Gender	Site of tumor	Clinical details	Coexisting condition	Follow-up (months)	Metastasis
Muhlemann *et al*(5)	59	M	Scapula	Unknown	Burn scar	Unknown	No
Rosen *et al*(1)	Unknown	Unknown	Unknown	Unknown	Lentigo maligna	Unknown	No
Walker and Walker (6)	78	F	Thigh	Unknown	Burn scar	Unknown	No
Akiyama *et al*(7)	55	M	Right lower leg	Pigmented macule	Burn scar	108	Yes
Alconchel *et al*(8)	46	F	Thigh	Unknown	Burn scar	Unknown	Yes
Pool *et al*(2)	70	M	Right medical canthus	Crusted black nodule	Unknown	12	No
	50	M	Left eyebrow	Brown-black nodule	Unknown	24	No
	44	F	Forehead	Brown-black nodule	Lentigo maligna	108	No
	47	M	Nose	Brown-black nodule	Unknown	12	No
Cutlan *et al*(18)	72	F	Shoulder	Nonpigmented lesion	Unknown	3	No
Dorić *et al*(19)	61	F	Left preauricular area	Darkly pigmented nodule	Unknown	Unknown	No
Satter *et al*(20)	63	F	Left leg	Gray macule	Unknown	31	No
	73	F	Left forearm	Flesh-colored papule	Unknown	27	No
Rongioletti *et al*(21)	94	M	Back	Purple-brownish nodule	Hidradenoma	8	No
Pouryazdanparast *et al*(22)	62	M	Left ear	Cutaneous lesion	Sunburn	9	No
Leonard *et al*(9)	68	M	Left temple	Blue-gray nodule	Solar keratosis	Unknown	No
Miteva *et al*(10)	82	M	Nose	Skin-colored papule	Unknown	Unknown	No
Amerio *et al*(12)	32	F	Right arm	Brown-black nodule	Unknown	Unknown	No
Present case	63	F	Left lateral canthus	Brown-colored spot	Solar keratosis	14	No

M, male; F, female.
